# Massive benthic litter funnelled to deep sea by flash-flood generated hyperpycnal flows

**DOI:** 10.1038/s41598-019-41816-8

**Published:** 2019-03-29

**Authors:** Martina Pierdomenico, Daniele Casalbore, Francesco Latino Chiocci

**Affiliations:** 1 Institute of Environmental Geology and Geo-Engineering, National Research Council (IGAG-CNR), Rome, Italy; 2 University of Rome “Sapienza”, Rome, Italy

## Abstract

Marine litter is an emerging environmental threat affecting all world’s oceans including the deep seafloor, where the extent of the phenomenon is still largely unknown. We report the spatial patterns of macro-litter distribution within the Messina Strait’s channels (Central Mediterranean), focusing on the transfer mechanisms responsible for its emplacement, a key information to better understand litter distribution. Litter is patchy but pervasive on all surveyed channels, reaching densities up to ~200 items/10 m, the highest reported for the deep sea until now. Litter is often arranged in large accumulations formed by hundreds of land-sourced items, mixed to vegetal and coarse-grained debris, indicating an emplacement from sedimentary gravity flows. Such impressive amount of litter can be explained by the superposition of a very efficient source-to-sink sedimentary transport and a strong urbanization of the coastal area. These findings point out that macro-benthic litter pollution is a major, often overlooked, threat for deep-sea ecosystems. Further explorations are thus required in similar marine settings to fully understand the magnitude of the problem, since they may represent the largest litter hotspots in the deep-sea.

## Introduction

Marine litter is one of the most pervasive and fast growing anthropogenic alteration of world’s oceans^1^, that has been documented in all marine environments^2^, from coastal surface waters^3^ to the most remotes areas such as poles^4^, oceanic islands^5^ and abyssal plains^6,7^. While great attention is actually given to plastic and microplastic debris^8,9^, either beached^10^ or floating in the oceans^11^, the distribution of macro-litter on the seafloor (the so-called benthic litter)^12^, especially for the deep waters, is still poorly known^13^. This is a considerable knowledge gap, since the deep seafloor is recognized as one of the largest depocenter for litter on Earth^7,14^, being the final collector of any debris coming from land-based sources^15^.

Particularly, the Mediterranean Sea, a semi-enclosed sea with large urban and industrial concentrations along its coasts, heavy maritime traffic and limited water exchange through the Strait of Gibraltar, is considered as one of the sites with the highest density of benthic marine litter worldwide^2^.

In this paper, we examined and quantified the spatial distribution of a specific type of litter, that we define marine municipal solid waste (hereafter called MMSW) in the Messina Strait, which is one of the most geologically active areas of the Central Mediterranean Sea^16^. Its peculiar geomorphic setting is characterized by a steep mountain range very close to the coast, drained by short and steep streams (locally named *Fiumara*) able to carry huge bedload during seasonal flash-floods occurring from years to decades^17^, as well as by high average seafloor gradients (10°) and a dense drainage network of submarine canyons^18^. Specifically, due to the lack of continental shelf, the seafloor is characterized by several channelized features that are spatially connected with the mouth of the steep river courses, favouring a strong source-to-sink sedimentary transport. Repetitive multibeam surveys performed a few kilometres south of the study area witnessed the evolution from subaerial flash floods to submarine hyperpycnal flows able to erode the seafloor^19^.

The above described setting, coupled with the densely populated coastal areas and poor disposal practices, thus represents a natural laboratory for the study of MMSW transfer to the deep sea. By the integrated analysis of Remotely Operated Vehicle (ROV) video observations, morpho-bathymetric and Side Scan Sonar (SSS) data, we are able to depict and describe an unexpected amount of benthic litter in these areas and discuss its distribution in terms of potential sources, transport pathways and mechanisms responsible for its emplacement.

## Results

### Geomorphology and seafloor characteristics of the Messina Strait canyons

**Figure 1:**
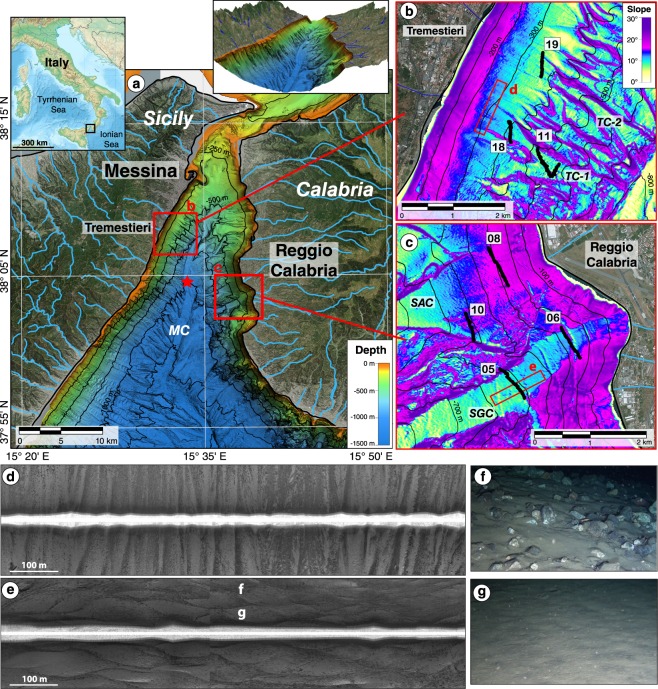
Data location and seafloor imaging. **(a**) Bathymetry of the Messina Strait (3D view in the upper-right inset) with fluvial drainage network (blue lines) and location of the dregde that recovered debris shown in Fig. 3h (red star). (**b,c**) Slope maps of the study areas on the Sicilian and Calabrian margins respectively, with location of ROV transects (black lines) and (**d**,**e**) SSS images (red rectangles). (**f**,**g)** ROV images (location in **e**) showing examples of coarse-grained (**f**) and sandy (**g**) sediment. MC – Messina Canyon; TC-1 – Tremestieri channel 1; TC-2 – Tremestieri channel 2; SAC – Sant’Agata channel; SGC – San Gregorio channel. The site maps (**a**–**c** and the upper-right inset) were generated with QGIS Version 2.18 (https://www.qgis.org/it/site). The satellite imagery was obtained from the Bing Aerial maps, using the QGIS *OpenLayers* plug-in (https://github.com/sourcepole/qgis-openlayers-plugin). The upper-left insert map is GEBCO relief data (downloaded from https://www.gebco.net) created using QGIS Version 2.18. The SSS images (**d**,**e**) were created with Caris Hips and Sips 6.1 software (http://www.caris.com/products/hips-sips/).

The overall physiography of the Messina Strait resembles that of a steep valley, where the offshore morphology prolongs the subaerial setting. A main axial canyon (the Messina Canyon, MC in Fig. 1a) runs along the whole strait, connecting the Sicilian (to the west) and the Calabrian (to the east) continental slopes. On both sides of the Strait, the steep submarine slopes are carved by a dense network of small erosive channels, often morphologically linked with the *fiumara* mouths, that convey into the Messina Canyon (Fig. 1a). These erosive features show a different distribution and morphology on the two margins.

**Figure 2:**
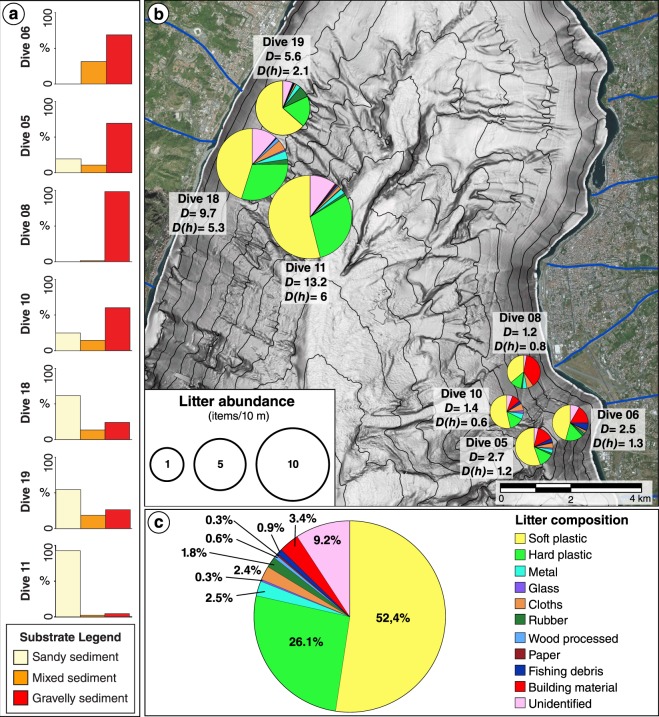
Seafloor characteristics, distribution and composition of marine litter. (**a**) Seafloor types and (**b**), categories of marine litter for each transect (location in Fig. 1). The size of the pie charts in (**b**) is proportional to litter abundance (D = total litter density and D(h) = litter density of heavy items, both expressed as items/10 m). (**c**) Overall composition of marine litter observed along all ROV dives in the Messina Strait canyons. The map in (**b**) was generated with QGIS Version 2.18 (https://www.qgis.org/it/site). The satellite imagery was obtained from the Bing Aerial maps, using the QGIS *OpenLayers* plug-in (https://github.com/sourcepole/qgis-openlayers-plugin).

On the Sicilian margin off Tremestieri village, the surveyed submarine channels (TC-1 and TC-2 in Fig. 1b) have an overall rectilinear course, a 150–300 m wide thalweg and an average spacing of about 500 m. They form headless channels at water depths of 300–400 m, where a marked decrease in slope gradients occurs at the base of a steep (up to 20°–25°) slope apron (Fig. 1b). On SSS records, the slope apron is characterized by high backscatter stripes parallel to the slope (Fig. 1d), that can be interpreted as a braided pattern of coarse-grained debris, distributed by sedimentary gravity flows. Immediately downslope, ROV dives show that sandy sediment prevails, especially in the deeper transect (Fig. 2a) with only small patches (of few squares meters) of cobbles and boulders.

On the Calabrian margin off Reggio Calabria, the setting is more complex, with some areas characterized by headless channels at about 400 m depth (i.e. the Sant’Agata channel, SAC in Fig. 1c) while other areas are morphologically dominated by fewer but larger channels, such as the San Gregorio channel (SGC in Fig. 1c). Notably, this channel is characterized by a 500 to 1200 m wide, flat-bottomed thalweg, originating from a large embayment on the coastal area (Fig. 1c).

The available SSS data along the thalweg of San Gregorio channel show the presence of a braided pattern of high backscatter zones, indicating the occurrence of coarse-grained gravity flows (Fig. 1e). ROV seafloor images collected across the SSS track confirm the presence of gravel, pebbles, cobbles and occasionally large boulders in correspondence of high backscatter areas (Fig. 1f), while sandy sediment is associated with low backscatter (Fig. 1g). Overall, ROV dives show that San Gregorio and Sant’Agata channels are dominated by gravelly sediment, especially on shallower dives (Fig. 2a).

### Spatial distribution and composition of Marine Municipal Solid Waste (MMSW)

Nearly 4000 litter items were counted along seven ROV transects, which explored a total linear distance of 6,420 m (Table 1). Average litter abundance per transect ranges from 1.2 items/10 m in Sant’Agata channel at ~300 m depth, to 13.2 items/10 m in Tremestieri channel 1 at 520–580 m depth (Table 1). Litter distribution is heterogeneous, with higher concentrations occurring within the Sicilian channels compared to the Calabrian ones (Fig. 2b). An overall increase in litter abundance is observed within each single channel with increasing depth, which in turn is associated with a decrease in slope gradients.

**Table 1:** ROV video transects location, length and water depth.

Location	Track	Coordinates				Depth range min - max (m)		Total length (m)	Analyzed length (m)	Litter items (n)	Litter abundance (items/10 m)	Litter size		
		Start		End										
		Latitude	Longitude	Latitude	Longitude							S	M	L
SGC	Dive-05	38°03′26.3″N	15°37′31.9“E	38°03′08.9“N	15°37′50.0“E	478	518	1051	924	253	2.7	139	92	22
SGC	Dive-06	38°03′32.6″N	15°38′31.4″E	38°03′50.7″N	15°38′17.5″E	243	276	818	784	192	2.5	106	64	22
SAC	Dive-08	38°04′37.8″N	15°37′24.1″E	38°04′15.3″N	15°37′38.9″E	275	314	1215	1161	141	1.2	95	36	10
SAC	Dive-10	38°03′44.2″N	15°37′13.2″E	38°03′59.5″N	15°37′07.1″E	508	524	701	516	71	1.4	40	25	6
TC-1	Dive-11	38°07′16.5″N	15°33′04.3″E	38°07′26.8″N	15°32′47.4″E	518	581	1778	1742	2298	13.2	1116	1066	116
TC-1	Dive-18	38°07′30.6″N	15°32′26.2″E	38°07′43.9″N	15°32′23.9″E	384	424	723	670	651	9.7	388	242	21
TC-2	Dive-19	38°08′13.0″N	15°32′50.4″E	38°08′28.4″N	15°32′51.9″E	337	381	722	623	351	5.6	213	102	36

The length of the analysed video, the number of litter items counted, their abundance and size class are also reported. SGC – San Gregorio channel; SAC – Sant’Agata channel; TC-1 – Tremestieri channel 1; TC-2 – Tremestieri channel 2. For litter size: S = small items <10 cm; M = medium items 10–50 cm; L = large items >50 cm.

**Figure 3:**
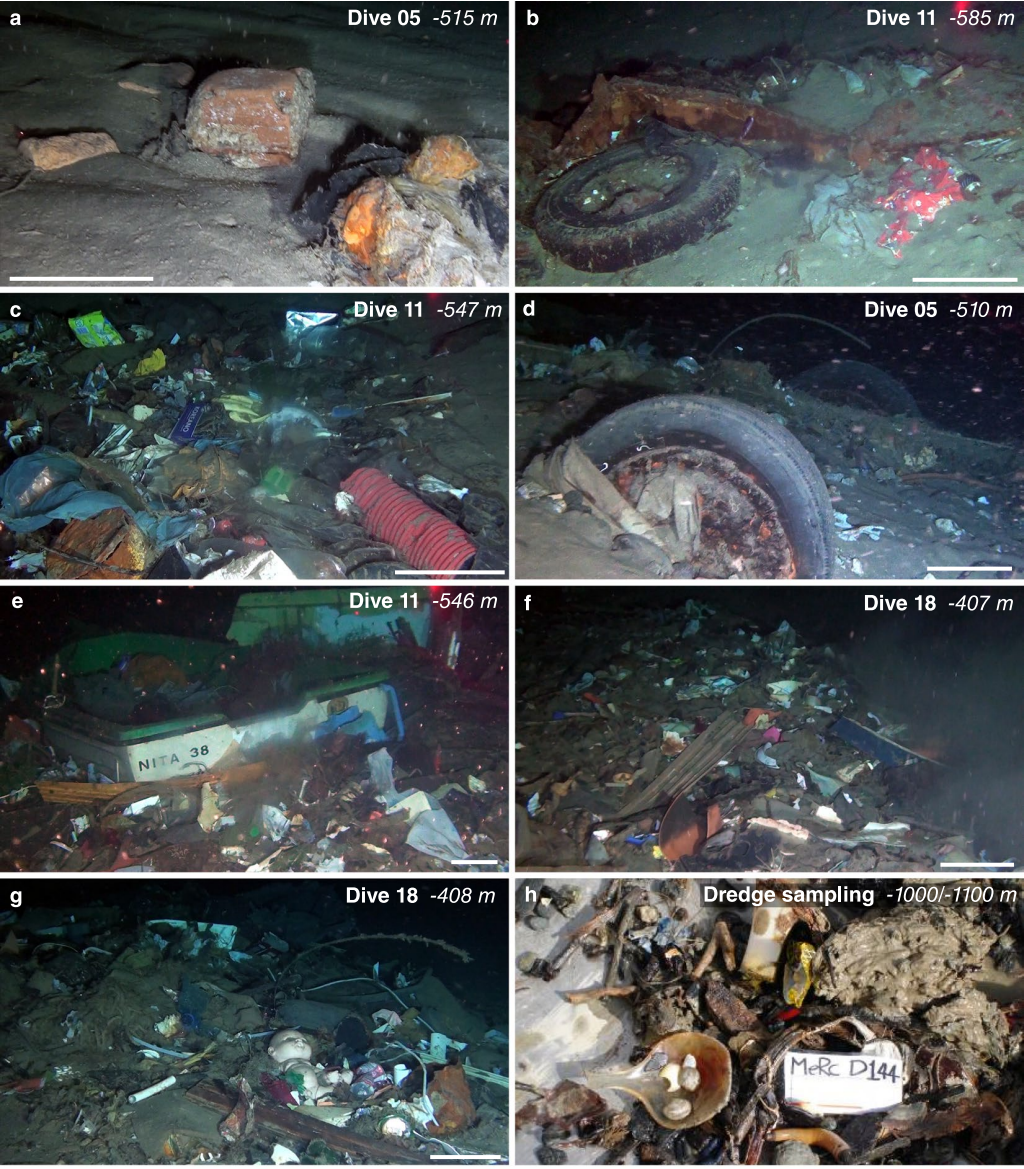
Benthic litter and MMSW mounds. **(a**–**g)** The photos extracted from ROV video footage show examples of benthic litter accumulations observed within channels in the Messina Strait, including: bricks (**a**), tires, clothes and large metallic objects (**b**), small (**c**) and large (**d**–**g**) MMSW mounds, sometimes formed around large items such as a buried car (**d**) and a sunken boat (**e**). White scale bar: 20 cm. (**h**) Litter recovered from a dredge performed within the axis of the Messina Canyon at 1000–1100 m depth (location in Fig. 1).

Regarding litter composition, soft plastic, essentially represented by plastic bags and packages, is by far the most widespread category, representing the 52.4% of the total litter debris, followed by hard plastic that accounts for 26.1% (Fig. 2c). This reflects the large global production stocks and consumption of plastic materials that usually are among the main components of municipal solid waste^15^. Although is not a trivial task to determine the exact source of marine litter, the typology of the items observed (Fig. 3) and the extreme paucity of fishing debris (0.9% of the total, Fig. 2c) suggest that most of the anthropogenic debris in the Messina Strait originates from land-based sources. For instance, common hard plastic items include bottles and cups, but also toys, sanitary napkins, corrugated and gutter pipes, garden hoses, electrical outlet boxes and even some windows shutters. Abundant building material (i.e. bricks, cement piles…, Fig. 3a) is also observed, especially on the Calabrian margin (Fig. 2b), as well as several cloths and foam paddings. Wood processed material is mostly represented by boards and fragments of wooden furniture, whereas metal litter frequently includes large objects such as plates and barrels (Fig. 3b). Moreover, the bad conservation status of litter items and their partial burial sometimes does not allow to identify its composition, that are therefore included in the class “Unidentified”, accounting for the 9.2% of the entire distribution (Fig. 2c).

Regarding the size, 53% of the observed litter is <10 cm, generally due to the fragmentation of larger objects, while 41% ranges between 10 and 50 cm (Table 1). Several large items (>50 cm) are mostly recognized within the Tremestieri and San Gregorio channels (Table 1). Noteworthy, a buried car (Fig. 3d) is found at 510 m in the San Gregorio channel, while four small boats (Fig. 3e) are observed in Tremestieri channel 1 between 580 and 520 m depth. One of those was identified as a small boat belonging to a touristic facility located in the Sicilian coast, about 40 km to the south of the head of Tremestieri channel 1.

**Figure 4:**
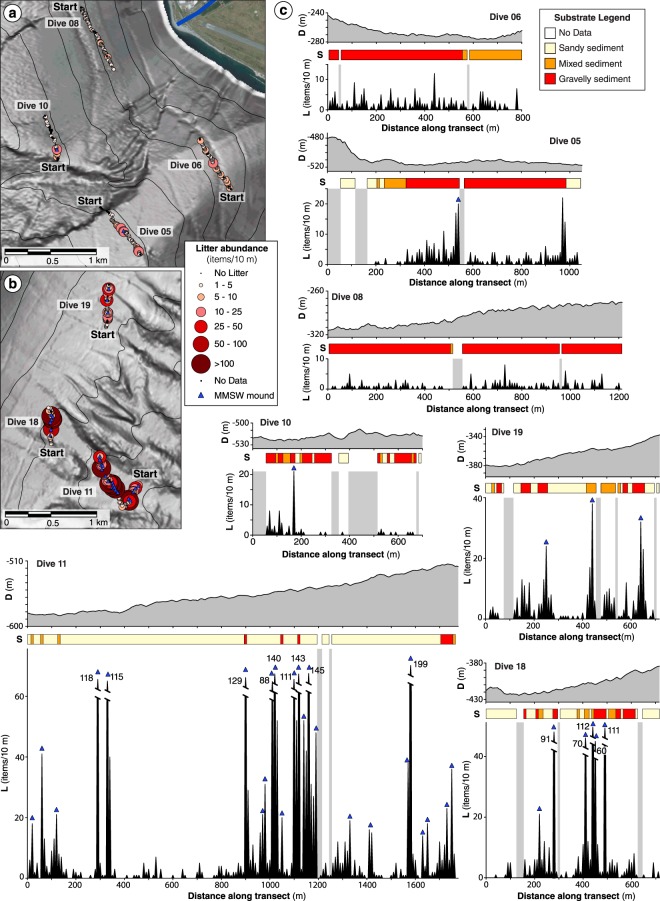
Small-scale distribution of benthic litter. **(a**–**c**) The maps (**a**,**b**) and the density plots (**c**) show variation of the benthic litter abundance along the video transects and the distribution of the MMSW mounds (blue triangles). In the plots in (**c**) the X axis indicates the distance along the track, expressed in meter from the origin of the transect, while the Y axis indicate the litter density (L), expressed in items/10 m. Changes in depth (D) and seafloor composition (S) along the tracks are also illustrated above the density plots. Note that vertical and horizontal scales are maintained for all graphs. The maps in (**a**,**b**) were generated with QGIS Version 2.18 (https://www.qgis.org/it/site). The satellite imagery was obtained from the Bing Aerial maps, using the QGIS *OpenLayers* plug-in (https://github.com/sourcepole/qgis-openlayers-plugin).

The detailed analysis of litter distribution along the ROV tracks (every 10 m) shows an overall high variability in the concentration of anthropogenic debris throughout the transects, except for the ROV Dive 08 on the slope apron upslope of Sant’Agata channel (Fig. 4a,c). Moreover, the litter abundance along the transect is much more uneven on the Sicilian side, varying of up to two orders of magnitude (from 1 to ~200 items/10 m), respect to the Calabrian side (Fig. 4c). The maximum concentration of litter was observed at or just after the confluence of channel branches in the Tremestieri channel 1 (Fig. 4b).

### MMSW mounds

Besides the large variability in litter abundance along most of the transects, ROV videos also show the presence of chaotic clusters of benthic litter accumulations that we define “MMSW mounds” (Fig. 3c–g). Particularly, these deposits have a well-defined boundary and a limited spatial extent (typically few square meters), are often elongated along the maximum slope and sometimes display a lobate shape (Fig. 3f). They are composed by a mixture of coarse sediment intermingled with light and heavy litter items that undoubtedly belong to municipal solid waste (including the most disparate and odd objects i.e. pieces of domestic appliances, chairs and tables, shoes, foam mattresses, dolls and toys, car mats, plates, electric wires, watering cans, plastic Christmas trees, toilet seats… Fig. 3c,e–g), and vegetal debris such as tree or *Posidonia oceanica* leafs and wood pieces. In some cases, MMSW mounds are found around large items such as semi-buried car and small boats, which act as a barrier for smaller debris (Fig. 3d,e). These anthropogenic deposits are widespread in the Tremestieri channels, including from tens to hundreds of litter items (up to 241 items in a single MMSW mound). On the contrary, large MMSW mounds are not found in the dives carried out on the Calabrian margin, where only small litter clusters (typically <2 m^2^) can be observed, being composed by up to few tens of items (Fig. 4c). MMSW mounds also include buried or partially buried items, so that the reported litter abundance is likely to be underestimated.

## Discussion

Nowadays, floating plastic and microplastics contamination of the oceans is rather well-known and is considered one of the world’s most pressing environmental concerns^11,20,21^. In this work we focused in a less-known and undervalued specific type of benthic marine litter pollution, strictly related to municipal solid waste mobilized during riverine floods and transferred into the deep sea via sedimentary gravity flows. Such phenomenon is likely to represent a significant threat for marine ecosystems in specific areas, whose distribution and characters at global scale are almost unknown^13^.

The role of submarine canyon as main carrier of marine litter from shallow water to the deep sea has been already highlighted by several authors^22–24^. However, the concentration of anthropogenic debris we found on the channel’s thalweg is impressive (Figs 2–4 and Table 1), as high as 13.2 items/10 m, averaged on a cross-thalweg transect (locally it can be hugely higher, up to ~200 items/10 m). If we extrapolate the observed litter abundances over an area of 1 km^2^ (the unit area most commonly reported in literature) we obtain minimum density values of 121,000 items/km^2^ and maximum of 1.3 million of items/km^2^ (Supplementary Table S1). This concentration is up to three orders of magnitude higher than the abundances reported for any submarine canyon of the world^7,22–27^ (Supplementary Table S1), globally considered as benthic litter hotspots^2^. Excluding the light litter items (soft plastic and paper), that could have potentially travelled long distances and could be easily transported by bottom currents, the abundance of heavy litter remains considerably higher (Fig. 2b), with respect to those reported from other submarine canyons of the Mediterranean Sea^7,22,24,25^ (Supplementary Table S1).

The study area has a rather peculiar physiographic setting (not uncommon however in the Mediterranean Sea) that causes the occurrence of violent flash-floods in the short but very steep ephemeral streams, locally called *fiumara*. This fact, coupled with the strong urbanization of the coastal sector and the paucity of proper solid waste management (that have made often possible the use of dry *fiumara* beds as waste disposal areas)^17^, may explain the astonishing amount of benthic litter and the emplacement of MMSW mounds in the submarine channels. More in detail, the submarine portion of the Messina Strait can be considered a giant composite canyon (Fig. 1a), with intense erosional and depositional processes strictly connected to subaerial drainage network^18,28^, resulting in a very efficient source-to-sink sedimentary transfer and possibility for land-sourced litter to be displaced into the deep sea.

Rivers are recognized as one of the main sources of marine litter worldwide and their transport capacity is strictly related to the runoff variability driven by seasonality^29,30^. In this regard, the semi-arid Mediterranean climate determines a very strong seasonal contrast. Indeed, the *fiumara* are characterized by extreme yearly variations in the discharge, resulting in dry beds in summertime and winter floods with values up to several hundreds of m^3^/s during short and severe rainy periods^31^. On river valleys these meteorological events are able to trigger widespread small-scale mass-wasting events mobilizing volumes up to hundreds of thousands of m^3^ of soil in very short timespans^32,33^, due to the proneness to mass-movement of the Calabrian and Sicilian slopes related to a combination of steep gradients and lithological factors^34^. As a consequence, variation in solid/liquid discharge ratio within *fiumara* may span from 0.004 for summer suspended load, to 0.5 for winter floods^31^. These almost-hyper concentrated floods may evolve into muddy/debris flows able to carry a large amount of coarse-grained sediment (including large and heavy blocks). Once they reach the coast, they can easily generate hyperpycnal flows in the marine environment due to their high density^35^. This is the case, for instance, of the 2009 flash-flood that struck the Sicilian coast near our study area, when dense (100 s kg/m^3^) and quick (estimated velocity of 10–20 m/s) debris flows^36^ caused submarine gravity flows, with seafloor erosion and slope failures in the offshore areas, mobilizing volumes of several tens of thousands of m^3^ of sediment^19^. It is noteworthy that such floods have a recurrence interval in the order of few decades in a given *fiumara*, resulting in a frequency of some years if averaged throughout the entire region^17^.

Once the debris (and litter) is funnelled in channels’ head by flash-flood generated hyperpycnal flows, it may be further re-mobilized by gravity flows acting within submarine channels. These events can be triggered by storms or floods^37^, or by the frequent earthquakes affecting the region^38^. In addition, sedimentary gravity flows are able to occur without major external triggers^39^.

In the study area, the witness of sedimentary gravity flows is the presence of high sonar backscatter zonation in the available SSS records, and the geometry of coarse-grained deposits observed from ROV dives. More in detail, the chaotic arrangement of the coarse-grained deposits (with large boulders >50 cm) mixed with vegetal debris and heavy litter items (including a buried car, furniture fragments and building material) let us to hypothesize a transport by sedimentary gravity flows, possibly driven by fast and dense basal layers, as recently observed in Monterey Canyon^39^. The occurrence of a dense basal layer would allow to carry material with different size, shape and density throughout rafting processes. Although no velocity data are available for sedimentary flows in the Messina Strait, a flow transit velocity >4 m/s for a turbidity current triggered by a submarine landslide at the nearby Gioia Canyon head (north of the Messina Strait) was estimated based on a cable breaking occurred in 1997^40^. Considering the steeper slopes of the Messina Strait channels, it is reasonable to hypothesize similar or even higher velocities for the sedimentary flows in the study area. The emplacement of the transported material should occur through en masse deposition from the flow, as suggested by the occurrence of lobate and downslope elongated MMSW mounds, and it seems to be mostly controlled by seafloor topography. Indeed, the higher concentration of MMSW mounds was found in correspondence of marked decrease in slope gradients as well as at the confluence of channel branches where flows are able to expand (Fig. 4).

Notwithstanding this efficient source-to-sink sedimentary transport, some differences can be noted between the two surveyed areas. For instance, the higher benthic litter abundance and presence of large MMSW mounds on the Sicilian side compared with the Calabrian side of the Messina Strait can be explained by:

- a higher population density in the Sicilian area with respect to the Calabrian area (data from ISTAT, National Institute of Statistics, https://www.istat.it), that would confirm the correlation between concentration of benthic litter and human population density^3,23,25,41^;
- the fact that we likely surveyed different morpho-sedimentary zones of the channels. Specifically, higher slopes and gravelly seafloor in the ROV dives along the Calabrian margin (Figs 1c and 2a) could indicate a by-passing area for sedimentary gravity flows. On the contrary, lower seafloor gradients associated with a predominant sandy seafloor in the ROV dives within the Sicilian channels (Figs 1b and 2a) could indicate an intra-slope temporary depositional area for these sedimentary gravity flows, whose final base level is the Messina Canyon.

The recovery of MMSW (i.e. shoes, kitchen utensils…) within dredges on the axial Messina Canyon at 1000–1100 m depth (Fig. 3h) is indeed a clear evidence that larger sediment-gravity flows are able to flush the entire tributary channels. These large events could be triggered by strong earthquakes that commonly struck this area due to its tectonic setting, such as the 1908 event. This earthquake was able to generate a turbidite flow that produced cable breaks up to 230 km far from the Messina Strait^42^, whose deposits were recovered in two cores at depth of 3814 and 3845 m, 20 km further southward from the breaks^43^.

It is clear that further work, including deeper and more extensive ROV and SSS explorations is needed to gain a better view of the overall MMSW distribution across- and along-slope in the Messina Strait. This knowledge coupled with post-event (earthquakes, flash-floods, storms…) surveys would be fundamental to constrain the controlling factors and residence time of benthic litter in this area.

More generally, the presented results indicate that densely populated coastal areas, lying on geologically-active settings where the morphology of subaerial and submarine slope is strictly linked, may led to the development of the largest benthic litter hotspots in the deep sea. This issue should be carefully considered by policy-makers, because it can extensively affect large seafloor sectors. This is particularly important considering that the location of seafloor debris accumulation areas still need to be precisely pinpointed^13^ and that the environmental consequences of this pollution are still poorly understood^44,45^. However, severe impacts can be figured out, own to the high vulnerability to anthropogenic drivers commonly attributed to deep-sea ecosystems^46^.

## Methods

### Geophysical data acquisition and processing

Multibeam bathymetry in the study area was collected during several oceanographic cruises carried out between 2005 and 2009 onboard the R/V *Universitatis*-CoNISMa, the R/V *Urania*-CNR and a small boat (*Calafuria*-CoNISMa) for shallow water surveys. Data were acquired with multibeam systems working at different frequency (50, 100 and 455 kHz) according to different bathymetric ranges. All surveys were positioned by DGPS and multibeam calibration was achieved by daily acquisition of sound velocity profiles and by patch test surveys. Moreover, real-time sound velocity close to the transducer was used as well as tide corrections provided by the nearby Messina tide-gauge station (www.mareografico.it). Raw data were processed using Caris Hips and Sips 6.1 software, encompassing data re-calibration and depth control, application of correct tide, statistical filters and manual editing of single swaths to remove out-of-sequence beams and spikes. Data were merged and gridded to obtain a single Digital Terrain Model (DTM) of the study area with a 10 m cell size.

Side Scan Sonar data were acquired during the oceanographic cruise RITMARE 2016, carried out from 11 to 24 February 2016 onboard the R/V *MinervaUno*-CNR, using an Edge Tech DF1000 system. The sonar system emitted a dual simultaneous frequency of 100 and 500 kHz with a resulting swath width of 200 m and was towed about 30 m above the seafloor at constant speed of 2–3 knots. Side Scan Sonar records were processed using Caris Hips and Sips 6.1 software. Data were radiometrically corrected to remove variable acquisition gains, power levels, insonification area and grazing angles, whereas geometric corrections were applied to compensate for the slant range. The final mosaic, with a 0.20 m horizontal resolution, was exported as a georeferenced TIF. The complex topography produced by canyons prevented from a complete sonar coverage within the study area, so that sonar profiles were collected only along the thalweg of San Gregorio and Sant’Agata channels (Fig. 1c), whereas on the Sicilian side of the Messina Strait SSS profiles parallel to the coastline along the slope apron were performed (Fig. 1b).

### ROV video collection and analysis

ROV dives were performed during the oceanographic cruises RITMARE 2016 and PASC (carried out from 15 October to 1 November 2016 onboard the R/V *MinervaUno*-CNR), using the remotely operated vehicle POLLUX III (GEI, maximum operative depth 600 m). The ROV was equipped with a digital camera (Sony CCD 1/3′′), a high definition camera (Sony HDR-HC7), ultra-short baseline positioning system (USBL) to ensure detailed records of the ROV tracks (accuracy ± 2 m) and two parallel lasers providing a reference scale of 20 cm on the videos. Survey sites were distributed along each side of the Messina Strait and ROV video transects were performed within the thalweg of the channelized features, following tracks perpendicular to the main axial channel.

Post-processing of USBL position data was performed to reduce uncertainty associated to the ROV navigation tracks. After manual removing of out-of-sequence beams and spikes, smoothing of the navigation tracks was performed.

The analyses of the video sequences included the description of the seafloor texture and the identification and enumeration of marine litter. Three classes of seafloor type were defined and mapped along the transects: sandy sediment, mixed sediment (corresponding to a dominant sandy seafloor with sparse gravel) and gravelly sediment (corresponding to a highly heterogeneous seabed formed by a sandy seafloor with interspersed gravel, pebbles and cobbles, with occasionally large boulders).

Marine litter larger than 2 cm was identified with a progressive distance from the beginning of the transect according to the ROV’s post-processed USBL position data. Litter was divided into categories (Fig. 2c) and sorted by size following the semi-quantitative scale of Mordecai *et al*.^23^, which defined three size classes: small litter including objects smaller than 10 cm, mid-size litter for debris between 10 and 50 cm, and large litter when object size exceeds 50 cm. Additionally, following the approach of Tubau *et al*.^24^, litter was quantified by its apparent weight and divided into two categories according to estimated density and associated floatability: light litter, including soft plastic and paper, and heavy litter. Sequences where silt clouds obscured the image or where the altitude of the ROV above the seafloor was too high to properly identify marine litter (>2 m) were considered of poor quality; the corresponding track length was quantified and excluded from subsequent analyses (light-grey areas in the histograms of Fig. 4). The length of the edited transects was then calculated and the total number of items for each dive was converted to items/10 m. To assess the small-scale distribution of marine litter each ROV transect was divided into successive fragments 10 m-long. Marine litter abundance, seafloor type and depth (extracted from the available bathymetric data) were reported for each transect fragment.

The main uncertainties in the estimate of litter density from ROV videos are related to the presence of buried items and to the impossibility to count all litter objects within MMSW mounds. Specifically, accumulation of litter items within mounds implies that some items can be hidden o partially buried underneath larger objects; furthermore, the field of view of the ROV camera above the MMSW mounds could not be appropriated to depict all objects within litter accumulations. The ROV vehicle strongly reduced its velocity in correspondence of the MMSW mounds and still photos with different fields of view were extracted from the videos to help identification of litter items and fragments. However, it is likely the occurrence of litter items not clearly visible and therefore not reported in the counting. Such uncertainties are not precisely quantifiable but may led to underestimations of the real amount of litter items.

To compare the abundance of litter in the Messina Strait with those reported in previous studies (Supplementary Table S1), linear litter densities were converted into items/km^2^. The area inspected during each dive was estimated by multiplying the transect length by the average width of vision of the ROV. This last parameter was tentatively determined using the camera laser scale from video, ranging between 0.80 and 1.2 m in edited sequences due to small variations in ROV altitude, so an average width of 1 m was assumed. Considering that areal litter density estimates are given in 1 km^2^ (the unit area most commonly reported in literature) and these were only used for rough comparison with previous studies taking into account the order of magnitude of litter abundance, the uncertainties in the estimate of surfaces covered by each track can be regarded as negligible. Moreover, it should be considered that the estimated densities are mostly referred to the thalweg of the channels, whereas few data are available for the flanks and surrounding continental slope, so the extrapolation of such densities to the inter-channel areas is not straightforward.

## Supplementary information

Supplementary Table S1
