# Supplementary material for: Massive benthic litter funnelled to deep sea by flash-flood generated hyperpycnal flows
Source: Sci Rep. 2019 Mar 29;9:5330. doi: 10.1038/s41598-019-41816-8 (PMC6441077; doi:10.1038/s41598-019-41816-8)
Supplement: Supplementary file 1 — Supplementary Table S1 [file 41598_2019_41816_MOESM1_ESM.docx]

Title

**Massive benthic litter funnelled to deep sea by flash-flood generated hyperpycnal flows**

Martina Pierdomenico, Daniele Casalbore, Francesco Latino Chiocci

**Supplementary Table S1. Worldwide benthic marine litter densities.** The abundance of benthic litter expressed as items/km^2^, the depth, geographic location and the method used for observation are also indicated. *density data from literature that were standardised to a uniform unit area of 1 km^2^. For densities reported from this study the values in brackets represent litter density of heavy items. ROV = remotely operated vehicle; TC = towed camera system; Trawl = Trawling gear; SUB = manned submersible

| **Geographic location** | **Depth** (m) | **Litter density** (items/km^2^) | **Method** | **Reference** |
| --- | --- | --- | --- | --- |
|  |  |  |  |  |
| **Continental margin off France (NW Mediterranean)** |  |  |  | *Galgani et al. (1996) |
| Gulf of Lion shelf, canyon, slope and bathyal plain | <200 - >1000 m | from 0 to 7.8 x 10^3^ | Trawl |  |
|  |  |  |  |  |
| **Continental margin of European Seas** | 50-2700 |  |  | *Galgani et al. (2000) |
| Baltic Sea | - | 1.3 x 10^2^ | Trawl |  |
| North Sea | - | 1.6 x 10^2^ | Trawl |  |
| Channel East | - | 0.2 x 10^2^ | Trawl |  |
| Bay of Seine | - | 0.7 x 10^2^ | Trawl |  |
| Celtic Sea (NE Atlantic) | - | 5.3 x 10^2^ | Trawl |  |
| Bay of Biscaye (NE Atlantic) | - | 1.4 x 10^2^ up to 5 x 10^3^ | Trawl |  |
| Gulf of Lion (NW Mediterranean) | - | 1.4 x 10^2^ | Trawl |  |
| NW Mediterranean | - | 1.9 x 10^3^ up to 1.01 x 10^4^ | Trawl |  |
| East-Corsica (Central Mediterranean) | - | 2.3 x 10^2^ | Trawl |  |
| Adriatic Sea (Mediterranean) | - | 3.8 x 10^2^ | Trawl |  |
|  |  |  |  |  |
| **Canyons, shelf and slope off California (NE Pacific)** |  |  |  | Watters et al. (2010) |
| Continental shelf and Ascension, Año Nuevo, Soquel, Monterey, and Carmel submarine canyons | 20 - 365 | from 3.2 x 10^2^ to 6.9 x 10^3^ | SUB |  |
|  |  |  |  |  |
| **Canyons of Portugese Margin (NE Atlantic)** |  |  |  | Mordecai et al. (2011) |
| Lisbon Canyon | 1602 | 6.6 · 10^3^ | ROV |  |
| Setubal Canyon | 2194 | 2.5 x 10^3^ | ROV |  |
| Cascais Canyon | 4574 | 1.1 x 10^3^ | ROV |  |
| Nazaré Canyon | 741 - 4385 | from 0 to 2 x 10^3^ | ROV |  |
|  |  |  |  |  |
| **Canyons, shelf and slope of NE Atlantic and Mediterranean** |  |  |  | *Pham et al. (2014) |
| Faroe-Shetland channel (NE Atlantic) | 657 | from 0.3 x 10^2^ to 1.9 x 10^2^ | TC |  |
| Norwegian margin (NE Atlantic) | 304 | 9.7 x 10^2^ | SUB |  |
| Dangeard and Explorer canyons (NE Atlantic) | 578 | 7.2 x 10^2^ | TC |  |
| Nazaré Canyon (NE Atlantic) | 3144 | 4.2 x 10^2^ | ROV |  |
| Lisbon Canyon (NE Atlantic) | 1602 | 6.6 x 10^3^ | ROV |  |
| Setubal Canyon (NE Atlantic) | 2194 | 2.5 x 10^3^ | ROV |  |
| Cascais Canyon (NE Atlantic) | 4574 | 3.2 x 10^3^ | ROV |  |
| Guilvinec Canyon (NE Atlantic) | 661 | 6.6 x 10^3^ | ROV |  |
| Whittard Canyon (NE Atlantic) | 2668 | 1.4 x 10^2^ | ROV-TC |  |
| Anton Dohrn Seamount (NE Atlantic) | 992 | 1.9 x 10^2^ | TC |  |
| Condor Seamount (Central Atlantic) | 258 | 1.4 x 10^3^ | ROV |  |
| Josephine Seamount (NE Atlantic) | 1455 | 5.7 x 10^2^ | ROV |  |
| Hatton Bank (NE Atlantic) | 706 | 1.9 x 10^2^ | ROV-TC |  |
| Rockall Bank (NE Atlantic) | 702 | 0.7 x 10^2^ | ROV-TC |  |
| Rosemary Bank (NE Atlantic) | 577 | 3.3 x 10^2^ | TC |  |
| Pen Duick Alpha/Beta Mound (NE Atlantic) | 534 | 2.5 x 10^2^ | ROV |  |
| Darwin Mounds (NE Atlantic) | 1007 | 9.7 x 10^2^ | ROV |  |
| Charlie Gibbs Fracture Zone (Central Atlantic) | 2300-2600 | from 0.4 x 10^2^ to 2.9 x 10^2^ | ROV |  |
| Wyville-Thomson Ridge (NE Atlantic) | 670 | 1.1 x 10^3^ | TC |  |
| Gulf of Lion (NW Med) | 85 | 0.4 x 10^2^ | Trawl |  |
| Blanes Canyon (NW Med.) | 1496 | 3.2 x 10^3^ | ROV |  |
| Gulf of Lion Canyons (NW Med.) | 510 | 0.4 x 10^2^ | Trawl |  |
| Artic continental slope | 2450 | 1.4 x 10^3^ | ROV-TC |  |
|  |  |  |  |  |
| **Canyons of Portugese Margin (NE Atlantic)** |  |  |  | *Oliveira et al. (2015) |
| São Vicente Canyon | 93 - 553 | from 5.8 x 10^2^ to 3.3 x 10^4^ | ROV |  |
|  |  |  |  |  |
| **Canyons of Catalan margin (NW Mediterranean)** |  |  |  | Tubau et al. (2015) |
| Cap de Creus Canyon | 156 - 1570 | from 2.3 x 10^3^ to 2.9 x 10^4^ | ROV |  |
| La Fonera Canyon | 212 - 1724 | from 0 to 1.7 x 10^5^ | ROV |  |
| Blanes Canyon | 860 - 1509 | from 6.7 x 10^2^ to 5.3 x 10^3^ | ROV |  |
|  |  |  |  |  |
| **Channels of the Messina Strait (Central Mediterranean)** |  |  |  | This study |
| San Gregorio Channel | 243 - 518 | from 2.5 (1.2) x 10^5^ to 2.7 (1.3) x 10^5^ | ROV |  |
| Sant'Agata Channel | 275 - 524 | from 1.2 (0.6) x 10^5^ to 1.4 (0.8) x 10^5^ | ROV |  |
| Tremestieri Channel 1 | 384 - 581 | from 9.7 (5.3) x 10^5^ to 1.3 (0.6) x 10^6^ | ROV |  |
| Tremestieri Channel 2 | 337 - 381 | 5.6 (2.1) x 10^5^ | ROV |  |
